# Soluble CD206 in metastatic renal cell carcinoma: Relation to clinical–biochemical parameters and patient outcome

**DOI:** 10.1002/ijc.35194

**Published:** 2024-09-25

**Authors:** Kasper Munch Lauridsen, Holger Jon Møller, Mie Wolff Kristensen, Niels Fristrup, Frede Donskov, Marianne Hokland, Morten Nørgaard Andersen

**Affiliations:** ^1^ Department of Biomedicine Aarhus University Aarhus Denmark; ^2^ Department of Clinical Biochemistry Aarhus University Hospital Aarhus Denmark; ^3^ Department of Clinical Medicine Aarhus University Aarhus Denmark; ^4^ Department of Oncology Aarhus University Hospital Aarhus Denmark; ^5^ Department of Oncology University Hospital of Southern Denmark Esbjerg Denmark; ^6^ Department of Molecular Medicine Aarhus University Hospital Aarhus Denmark; ^7^ Department of Hematology Aarhus University Hospital Aarhus Denmark

**Keywords:** biomarker, CD206, macrophages, renal cell carcinoma, soluble CD206

## Abstract

The mannose receptor (MR/CD206) is a marker of M2‐like tumor‐associated macrophages. Membrane CD206 can be shed, releasing the receptor as a soluble protein (sCD206), which can be measured in serum. Here, we investigated the biomarker potential of sCD206 in patients with metastatic renal cell carcinoma (mRCC). Serum sCD206 was measured by an enzyme‐linked immunosorbent assay in 88 mRCC patients and 20 healthy controls (HCs). At diagnosis, serum sCD206 was elevated in patients with intermediate‐risk mRCC according to the Memorial Sloan Kettering Cancer Center (MSKCC) risk score, compared to both HCs and patients with favorable MSKCC risk score. Furthermore, sCD206 levels correlated with both sCD163 and C‐reactive protein. Soluble CD206 levels decreased after treatment initiation (*p* < .0001 at 5 weeks) but with a tendency toward elevated levels at time of progression, compared to baseline (*p* = .06). In univariate survival analysis, high levels of serum sCD206 at baseline was a significant risk factor associated with reduced overall survival (hazard ratio [HR] = 1.37, 95% confidence interval: 1.12–1.67, *p* = .002). Stratified by clinical risk scores, increased sCD206 was still a statistically significant risk factor of overall mortality (*p* < .01) in the intermediate‐risk group by both the MSKCC (HR = 1.48) and the newer International Metastatic RCC Database Consortium (IMDC) score (HR = 1.53). Furthermore, addition of sCD206 as a dichotomized variable to the IMDC risk score enabled separation of the intermediate‐risk group into two groups with survival comparable to those with favorable and poor risk, respectively. Overall, sCD206 is a potential add‐on biomarker for mRCC patients in the intermediate‐risk group of the current clinical risk scores.

## Graphical abstract

What's new?

Patients with metastatic renal cell carcinoma (mRCC) show highly heterogeneous treatment response and survival. Here, the authors demonstrate the potential prognostic value of the soluble form of the macrophage mannose receptor (sCD206) in patients with mRCC. High serum levels of sCD206 were associated with decreased overall survival in the intermediate‐risk groups identified using the MSKCC and the IMDC risk scores. Soluble CD206 can be measured in serum by standardized immunochemical assays and may become an important prognostic biomarker, if validated in independent cohorts.

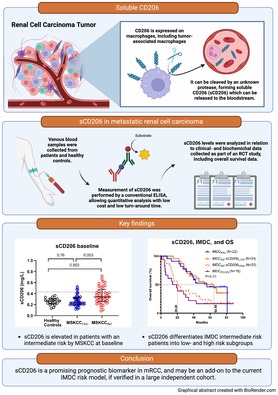

## 1 INTRODUCTION

Kidney cancer is a common disease with 342,000 annual cases and 131,000 deaths worldwide.^1^ The most common type of kidney cancer is clear cell renal cell carcinoma (RCC), representing approximately 80% of all cases.^2^ Metastatic RCC (mRCC) is common; 25%–30% of patients have metastases at the time of diagnosis, and further 20%–30% develop metastatic disease after primary nephrectomy, hence contributing to the high mortality rate.^3^, ^4^

In the past decades, medical treatment of mRCC has evolved dramatically with the introduction of immune and targeted therapies, including anti‐VEGF antibodies, tyrosine kinase inhibitors (TKIs), and immune‐checkpoint inhibitors.^2^ However, mRCC patients demonstrate high heterogeneity in relation to treatment response and survival, and thus clinical risk scores used to predict overall survival (OS) have been established, primarily the Memorial Sloan Kettering Cancer Center (MSKCC) and the International Metastatic RCC Database Consortium (IMDC) risk scores.^5^, ^6^ We recently reported that the macrophage‐activation biomarker soluble CD163 (sCD163) was an independent prognostic biomarker of OS in mRCC patients, and demonstrated its potential as an add‐on biomarker for IMDC intermediate‐risk mRCC patients.^7^

Here, we report the first analyses on the macrophage‐activation biomarker, soluble CD206 (sCD206) in mRCC patients. The macrophage mannose receptor, CD206, is expressed on some types of macrophages, dendritic cells, and on endothelial cells. It is involved in receptor‐mediated endocytosis through binding to carbohydrate structures.^8^ Increased expression of CD206 on macrophages has been demonstrated after stimulation with interleukin‐4 (IL‐4), IL‐13, and granulocyte‐macrophage colony‐stimulating factor *in vitro*,^9^, ^10^ and CD206 is considered a marker of tumor‐associated macrophages (TAMs).^11^ Due to cleavage of the membrane‐associated protein by a still unknown protease, CD206 is present in serum in a soluble form.^12^, ^13^ Elevated levels of sCD206 have been described in a few cancers, such as gastric cancer and multiple myeloma,^14^, ^15^ but serum levels in mRCC patients have not previously been studied. Thus, the aim of the present study was to describe levels of sCD206 in mRCC patients, including changes during treatment, and to evaluate the potential of sCD206 as a prognostic biomarker.

## 2 MATERIALS AND METHODS

### 2.1 Patients and healthy controls

The patients and healthy controls (HCs) included in the present study have previously been described.^7^, ^16^ In short, a total of 88 patients with mRCC, either MSKCC favorable (MSKCC_FAV_, *n* = 47) or intermediate (MSKCC_INT_, *n* = 41) risk score, were included from the phase II clinical trial (Danish Renal Cancer Group (DaRenCa) Study‐1), where patients were randomized to IL‐2/interferon‐α ± bevacizumab. The clinical outcomes of this study have been reported elsewhere.^16^ Blood samples were collected at baseline, 5 weeks, 9 months, and/or at progression. Based on clinical information from the phase II study, IMDC risk scores were calculated post hoc, as described by Heng et al.^6^ Total calcium and albumin were utilized to calculate corrected calcium by the following formula: Corrected calcium [mmol/L] = 0.02 × (normal albumin (40 g/L)—measured albumin) + total calcium. For calculation of the IMDC risk score, a corrected calcium of 2.55 mmol/L was used as a cut‐off for dichotomization. To summarize the total metastatic burden, a score was calculated by adding the number of organs affected by metastases to a sum.

Anonymized blood samples from 20 age‐ and sex‐matched HCs from the local blood bank, Department of Clinical Immunology, Aarhus University Hospital, Aarhus, Denmark were included as a control group.^7^

### 2.2 Measurement of sCD206, sCD163, and C‐reactive protein

Serum concentrations of sCD206 were analyzed at the Department of Clinical Biochemistry, Aarhus University Hospital, Aarhus, Denmark, by an in‐house sandwich enzyme‐linked immunosorbent assay, essentially as previously described^12^ in which a 95% reference interval of 0.10–0.43 mg/L was established based on 217 healthy adults.^12^ In the present study, one observation in the HCs of sCD206 was an extreme outlier (2.354 mg/L; nearest adjacent value 0.366 mg/L) and this observation was excluded from the data analysis (the result was confirmed by repeated measurements). sCD163 and C‐reactive protein (CRP) baseline data have previously been reported, including a description of the methods used for measurement.^7^

### 2.3 Statistical analysis

The distribution of continuous variables was assessed by QQ plots before analysis. Non‐Gaussian distributed variables were log‐transformed and re‐evaluated for Gaussian distribution. Comparison of continuous variables between two groups was performed by Student's *t*‐test if the distribution was Gaussian and variances were equal (by variance ratio test), otherwise Mann–Whitney *U* test was performed. Comparison of continuous variables between three or more groups was performed by one‐way ANOVA, if the distribution were Gaussian and variances were equal (by Bartlett's test), otherwise Kruskal–Wallis test was performed. Comparisons of categorical data between two groups were performed by *χ* ^2^ or Fisher's exact test (for outcomes with less than 5 events). Correlations were assessed using Pearson correlation.

Dynamics of sCD206 during follow‐up are shown as relative changes in percentages $\left(\frac{x-\mathrm{ref}}{\mathrm{ref}}\times 100\right)$ with the baseline concentration as the reference value. Statistical analysis of changes in sCD206 concentrations during treatment were analyzed directly by paired Student's *t*‐test (two‐way ANOVA or mixed‐effects models were not used due to missing data).

Survival analysis was performed by both Kaplan–Meier plots with logrank tests and by Cox proportional hazards regression analysis (Cox regression). sCD206 was analyzed as both a continuous and dichotomized variable by univariate Cox regression. When analyzed as a continuous variable, a factorization was incorporated such that the estimated hazard ratios (HRs) denote the relative risk associated with a sCD206 increase of 0.1 mg/L. Martingale residuals were analyzed as a function of sCD206 values, and a model including the squared function of sCD206 was utilized to reject a nonlinear relationship between sCD206 and ln(OS).

Effect modification, by MSKCC and IMDC risk score, on the prognostic value of sCD206 was analyzed by interaction analysis. Due to a trend for MSKCC (*p* = .08), and a significant interaction for IMDC (*p* = .047), effect modification was assumed present. Hence, to deal with the issue of effect modification, stratified HRs were calculated for subgroups of MSKCC and IMDC.

OS was calculated from the date of randomization until death or last follow‐up.

A two‐sided *p* < .05 was considered statistically significant. Prism 9 for Windows (GraphPad Software, San Diego, CA) was used to create graphs and Kaplan–Meier plots. STATA v. 15 for Mac or Windows (StataCorp LLC, TX) was used for the statistical analyses.

## 3 RESULTS

### 3.1 Baseline characterization of patients and HCs

Patients and HCs were matched by age (*p* = .20) and sex (*p* = 1.00).

The baseline patient characteristics have been reported and described in detail elsewhere,^7^ and are shown here with stratification by the median sCD206 concentration (Table 1).

**TABLE 1:** Baseline clinical and paraclinical data of included patients.

Variable	All Patients	sCD206 at baseline		
		Low	High	*p* Value
*N*	88	43	45	
Sex—*N* (%)				.06
Female	22 (25)	7 (16)	15 (33)	
Male	66 (75)	36 (84)	30 (67)	
Age—median (range)	57 (49–62)	56 (49–61)	58 (49–63)	.38
MSKCC—*N* (%)				**.003**
Favorable	47 (53)	30 (70)	17 (38)	
Intermediate	41 (47)	13 (30)	28 (62)	
IMDC—*N* (%)^a^				**.04**
Favorable	22 (26)	15 (35)	7 (16)	
Intermediate	46 (53)	23 (53)	23 (53)	
Poor	18 (21)	5 (12)	13 (30)	
Best response—*N* (%)				**.02**
Complete or partial response	36 (41)	22 (51)	14 (31)	
Stable disease	36 (41)	18 (42)	18 (40)	
Progressive disease	16 (18)	3 (7)	13 (29)	
Previous nephrectomy—*N* (%)	75 (85)	39 (91)	36 (80)	.23
Metastases—*N* (%)				
Lung	70 (80)	34 (79)	36 (80)	.91
Lymph node	55 (63)	28 (65)	27 (60)	.62
Liver	14 (16)	3 (7)	11 (24)	**.04**
Bone	23 (26)	10 (23)	13 (29)	.55
Soft tissue	17 (19)	9 (21)	8 (18)	.71
Adrenal glands	13 (15)	8 (19)	5 (11)	.32
Sites of metastases—*N* (%)^b^				.66
1	22 (25)	12 (28)	10 (22)	
2	30 (34)	14 (33)	16 (36)	
3	22 (25)	12 (28)	10 (22)	
≥4	14 (16)	5 (12)	9 (20)	
Smoking—*N* (%)				.32
Never smoked	29 (33)	13 (31)	16 (36)	
Former smoker	41 (47)	23 (55)	18 (40)	
Current smoker	17 (20)	6 (14)	11 (24)	
Paraclinical—median (IQR)				
Lactate dehydrogenase (mg/L)	168 (145–196)	167 (146–178)	176 (142–201)	.27
Hemoglobin (mmol/L)	8.3 (7.5–9.1)	8.6 (8.0–9.2)	8.0 (7.2–8.7)	**.02**
Total corrected calcium (mmol/L)	2.38 (2.30–2.50)	2.34 (2.28–2.44)	2.43 (2.30–2.52)	**.02**
Albumin (g/L)	38 (35–41)	39 (36–42)	38 (35–39)	**.02**
C‐reactive protein (mg/L)	9.84 (2.53–41.25)	3.18 (1.48–10.57)	24.00 (6.20–77.23)	**<.001**
Neutrophils (10^9^/L)	4.36 (3.17–5.66)	4.13 (2.98–5.50)	4.59 (3.41–6.44)	.14
Monocytes (10^9^/L)	0.63 (0.51–0.85)	0.59 (0.45–0.77)	0.68 (0.58–0.92)	**.01**
Platelets (10^9^/L)	272 (231–355)	248 (222–305)	300 (241–370)	**.02**
Soluble CD163 (mg/L)	2.18 (1.78–3.05)	2.01 (1.65–2.54)	2.34 (1.97–3.92)	**.006**

*Note*: Binary and categorical data are shown as *N* (numbers) with the percentage of the total cohort of patients in parentheses. Continuous data are shown as median values and interquartile range (IQR). Furthermore, the same parameters are shown after stratification of the patients by the median value of sCD206 (≥0.301 mg/L). *p* values were calculated from the relevant statistical test between the low versus high sCD206 groups. Statistically significant *p* values are highlighted by bold font. The present cohort does not include patients with MSKCC poor risk score, since such patients were excluded from the DaRenCa‐1 study.

Abbreviations: IMDC, International Metastatic RCC Database Consortium; MSKCC, Memorial Sloan Kettering Cancer Center.

^a^ IMDC scores could not be calculated in two cases due to missing data on total corrected calcium.

^b^ Calculated by organ systems affected.

No difference in age was observed, but a trend toward higher sCD206 among female patients was present (*p* = .06). Smoking status was not related to sCD206 levels (*p* = .32).

For both the MSKCC and the IMDC risk scores, there was a significant relationship between high sCD206 levels and more severe risk scores (*p* = .003; *p* = .04).

Looking at the best clinical response to treatment, more patients with progressive disease had high baseline sCD206, and more patients with a response to treatment had low baseline sCD206 (*p* = .02). Metastases in the liver were significantly associated with high levels of sCD206 (*p* = .04), otherwise no relationship between sCD206 and the tumor distribution or sites of metastases was observed. Lactate dehydrogenase and neutrophils are implemented in the MSKCC and the IMDC risk scores, respectively. However, these parameters were not statistically associated with sCD206 levels. The other biochemical biomarkers of these risk scores, hemoglobin (decreased), total corrected calcium (increased), and platelets (increased) were all associated with high levels of sCD206 (*p* = .02 for all). High sCD206 was also associated with higher monocyte (*p* = .01), CRP (*p* < .001), and sCD163 (*p* = .006) levels. Furthermore, a small decrease in albumin levels (*p* = .02) was associated with high sCD206.

### 3.2 Baseline levels of sCD206 and correlation to other biomarkers

**FIGURE 1:**
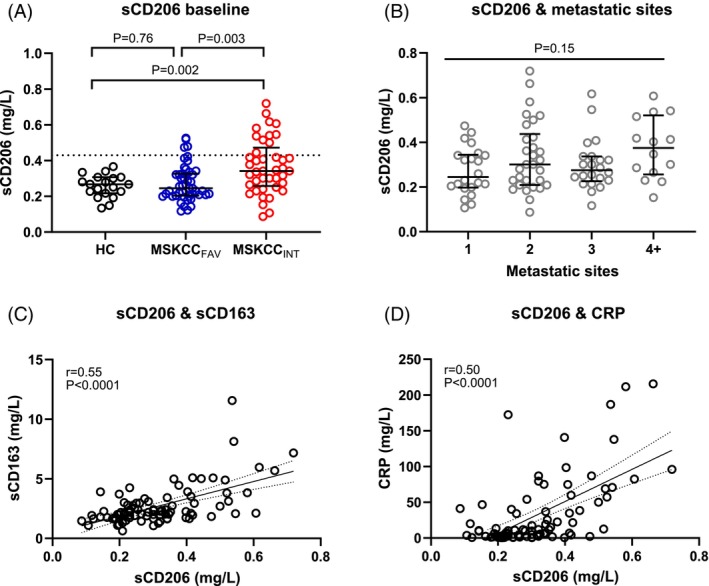
Soluble CD206 levels in patients and healthy controls. (A) Baseline levels of sCD206 of HCs and mRCC patients with an MSKCC_FAV_ or MSKCC_INT_ risk score measured by ELISA. Dotted line = 0.43 mg/L equal to the upper reference limit (see Rødgaard‐Hansen et al.^12^). (B) Baseline levels of sCD206 in mRCC stratified by numbers of organs affected by metastasis. (C) Correlation of sCD206 and sCD163 in mRCC patients. *p* and *r* values by Pearson correlation using ln(sCD206) and ln(sCD163). (D) Correlation of sCD206 and CRP in mRCC patients. *p* and *r* values by Pearson correlation using ln(sCD206) and ln(CRP). (A, B) Error bars show the median with IQR. (C, D) The best‐fitted line is shown based on non‐transformed values (with 95% CI as dotted lines). CRP, C‐reactive protein; ELISA, enzyme‐linked immunosorbent assay; HCs, healthy controls; IQR, interquartile range; mRCC, metastatic renal cell carcinoma; MSKCC, Memorial Sloan Kettering Cancer Center risk score; MSKCC_FAV_, favorable risk by MSKCC; MSKCC_INT_, intermediate‐risk by MSKCC; sCD206, soluble CD206.

Baseline concentrations of sCD206 for the mRCC patients (stratified by MSKCC) and HCs are shown in Figure 1A. sCD206 was not increased in mRCC compared to HCs when including all patients (*p* = .11). Stratified by risk groups, this was still the case for the MSKCC_FAV_ versus HCs (*p* = .76) whereas the MSKCC_INT_ patients had elevated sCD206 levels compared to both HCs (*p* = .002) and the MSKCC_FAV_ group (*p* = .003). The largest difference in medians was observed between the MSKCC_FAV_ and the MSKCC_INT_ groups, with a 0.97 mg/L higher median concentration in the MSKCC_INT_ group.

A possible association between the number of organs with metastasis and increasing levels of sCD206 was also investigated with sCD206 as a continuous variable; no statistically significant association was observed (*p* = .15; Figure 1B). Levels of sCD206 showed a statistically significant positive correlation in mRCC patients with both sCD163 and CRP (both *p* < .0001; Figure 1C,D).

### 3.3 Dynamics of serum sCD206 during treatment

**FIGURE 2:**
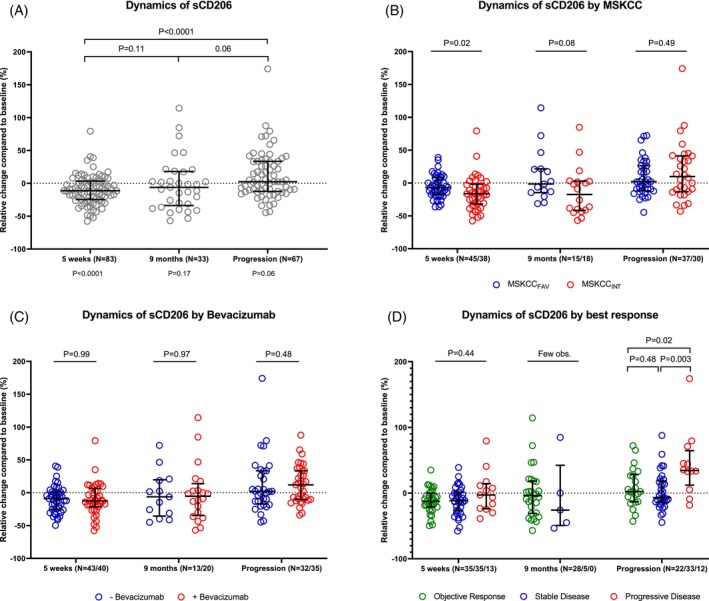
Dynamics of sCD206 during treatment. Dynamics of sCD206 as relative change (%) compared to baseline levels. (A) All patients. *p* values by paired Student's *t*‐test performed on absolute ln‐transformed sCD206 values. *p* values below the *x*‐axis show a comparison with the baseline. (B) As in (A), but stratified by the MSKCC risk groups. *p* values show a comparison of MSKCC groups at the different time points using relative change values. (c) Stratified by randomization to treatment with/without bevacizumab. *p* values show a comparison of intervention groups at different time points using relative change values. (D) Stratified by best clinical response. Objective response includes both complete and partial response. *p* values show comparison of the different groups by their best response at the different time points using relative change values. Statistical analysis was not performed at 9 months due to too few observations in the stable‐ and progressive‐disease groups. (A–D) Error bars equal median and interquartile range. MSKCC, Memorial Sloan Kettering Cancer Center risk score; sCD206, soluble CD206.

The relative change of sCD206 at 5 weeks, 9 months, and/or at progression, compared to baseline levels, was calculated (Figure 2). When investigating the entire cohort of mRCC patients (Figure 2A), a statistically significant decrease was observed at 5 weeks (*p* < .0001). At both 9 months (*p* = .17) and at the time of progression (*p* = .06), there was no statistically significant difference compared to baseline values. However, a trend toward increased values at the time of progression compared to baseline was observed. Compared to the lower values at 5 weeks, a significant increase was observed at progression (*p* < .0001).

The dynamics were then investigated after MSKCC stratification (Figure 2B). Here, at 5 weeks, there was a relative decrease from the baseline of 16.4% in the MSKCC_INT_ and 7.0% in the MSKCC_FAV_ groups, respectively, with a median difference in relative changes between the two groups of 9.4% (*p* = .02). The same tendency was observed at 9 months (*p* = .08), but no obvious difference was found at the time of progression (*p* = .49).

Next, the effect of treatment with bevacizumab was investigated, however, with no indication that this affected the dynamics of sCD206 during treatment (Figure 2C). Finally, associations between best clinical response and sCD206 dynamics were analyzed (Figure 2D). Patients with progressive diseases as their best clinical response had higher relative increase in sCD206 at progression, compared to patients with objective response (*p* = .02).

Thus, an initial reduction in sCD206 levels was observed upon treatment, which was more prominent in the MSKCC_INT_ risk group. A rebound toward baseline levels, or higher, was observed upon progression.

### 3.4 Serum sCD206 as a prognostic biomarker: Kaplan–Meier analyses

**FIGURE 3:**
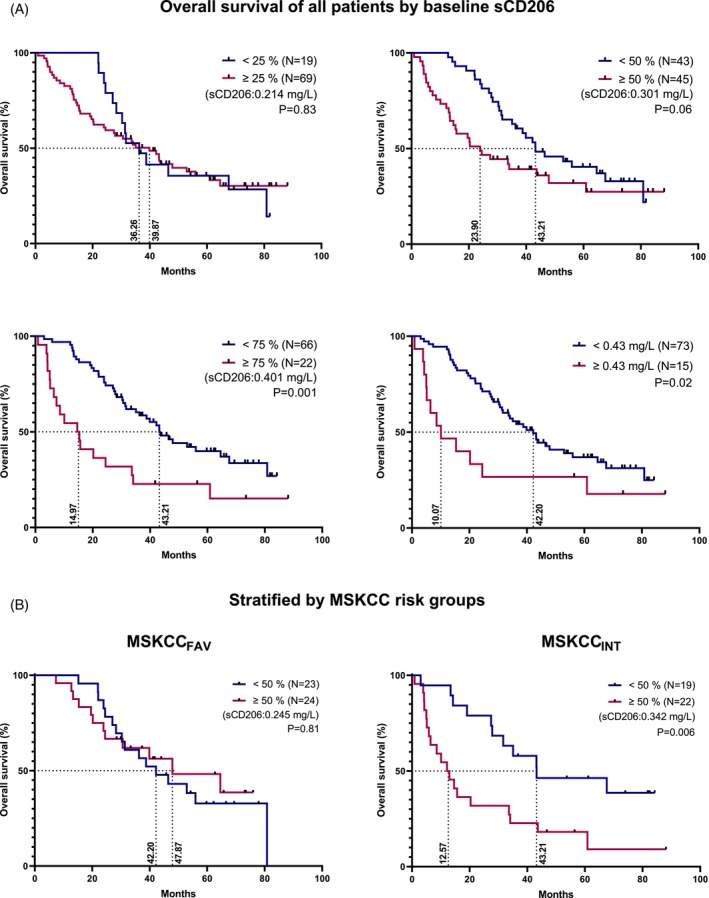
Survival analysis of mRCC patients by baseline sCD206. (A) Patients were dichotomized into two groups by sCD206 baseline values using the 25th, 50th, 75th percentiles, and the upper reference value of sCD206 (0.43 mg/L, see Rødgaard‐Hansen et al.^12^). (B) Patients were stratified by MSKCC risk groups, before being dichotomized based on the sCD206 50th percentile. (A, B) *p* values by log rank test. The separator value for binomial categorization is stated in parentheses on each plot. Censored patients are annotated with a rectangle. Dotted lines including numbers written vertically on the x‐axis indicate median OS. MSKCC, Memorial Sloan Kettering Cancer Center risk score; sCD206, soluble CD206.

The prognostic impact of sCD206 in mRCC was initially evaluated by Kaplan–Meier plots. First, all patients were included in the analyses. The sCD206 levels corresponding to the 25th, 50th, 75th percentile, and the upper reference value of 0.43 mg/L, respectively, were used as a cut‐off to perform binomial categorization of the patients into sCD206 low versus high groups (Figure 3A). Except for the 25th per‐ centile cut‐off, a clear tendency was observed towards high sCD206 levels at baseline being associated with worse outcomes, which was statistically significant when using the 75th percentile (*p* = .001) and the upper reference limit (*p* = .02) as the cut‐off. The median OS of the sCD206‐high group decreased with increasing sCD206 as cut‐off, illustrated by a median OS of 23.9 months and 10.1 months in the sCD206‐high when using the 50th percentile (0.301 mg/L) and the upper reference limit, respectively. The largest difference in median OS between low versus high sCD206 groups was observed using the upper reference limit, with median OS of 42.2 and 10.1 months, respectively.

Second, survival analyses were performed with cut‐offs as above, but after stratification by MSKCC or IMDC risk groups. The results for the 50 percentile cut‐off, stratified by MSKCC, are shown in Figure 3B, while the remaining graphs can be seen in Figures S1 (MSKCC) and S2 (IMDC). Overall, there were no significant differences in OS for the favorable group by either MSKCC or IMDC, while for the intermediate group of both MSKCC and IMDC, there was a tendency toward a reduced OS with higher sCD206 levels at baseline. A statistically significant difference in OS was observed when using the 50th percentile as the cut‐off for both MKSCC and IMDC intermediate‐risk groups. In the IMDC_POOR_ group, a tendency toward reduced OS by high sCD206 levels was also observed, although not statistically significant. However, both the favorable and poor IMDC risk groups had few observations (*N* = 22 and 18, respectively).

Thus, in the present cohort, sCD206 was a prognostic biomarker of OS in mRCC patients, most prominently in the intermediate‐risk groups.

### 3.5 Survival analyses by Cox regression

In a univariate analysis of all patients, sCD206 as a continuous variable (estimates based on 0.1 mg/L changes) was associated with an increased HR of overall mortality (HR = 1.37, 95% confidence interval [CI]: 1.12–1.67, *p* = .002). This was also the case when analyzed as a dichotomized variable, both when patients were split by the median (sCD206 ≥ 0.301 mg/L: HR = 1.94, 95% CI: 1.2–3.3, *p* = .01) and the upper normal reference limit (sCD206 ≥ 0.43 mg/L: HR = 2.13, 95% CI: 1.1–4.0, *p* = .02).

Effect modification between sCD206 and MSKCC or IMDC risk scores was analyzed, and a trend was found for MSKCC (*p* = .08), while a significant interaction was found for IMDC (*p* = .047). Due to this, stratified HRs were calculated for each stratum of the MSKCC and IMDC risk scores. Stratified by MSKCC, high sCD206 was associated with an increased HR in the intermediate‐risk group (HR = 1.48, 95% CI: 1.15–1.91, *p* = .002); however, no association was found in the favorable risk group (HR = 1.04, 95% CI: 0.67–1.61, *p* = .86) (see Table 2 for all results). Stratified by IMDC, sCD206 was again associated with an increased HR in the intermediate‐risk group (HR = 1.53, 95% CI: 1.15–2.04, *p* = .003), but no association was found in the favorable (HR = 0.51, 95% CI: 0.20–1.30, *p* = .16) or the poor risk group (HR = 1.19, 95% CI: 0.84–1.70, *p* = .33) (see Table 2 for all results). However, few patients were present in the favorable (*N* = 22) and the poor (*N* = 18) risk groups.

**TABLE 2:** Hazard ratios by Cox regression of sCD206 in relation to OS.

Risk score	*N*	HR	95% CI	*p* Value
All patients				
	88	1.37	1.12–1.67	0.002
Stratified by MSKCC				
Favorable	47	1.04	0.67–1.61	0.86
Intermediate	41	1.48	1.15–1.91	0.002
Stratified by IMDC				
Favorable	22	0.51	0.20–1.30	0.16
Intermediate	46	1.53	1.15–2.04	0.003
Poor	18	1.19	0.84–1.70	0.33

*Note*: Hazard ratios (HR) of soluble CD206 (sCD206) was calculated using Cox proportional hazards regression. The clinical outcome was overall survival (OS), and sCD206 was included as a continuous variable with HRs calculated based on a 0.1 mg/L increase. First, a univariate HR was calculated for all included patients based on the baseline levels of sCD206. Afterward, HRs were calculated in subgroups based on stratification by either the Memorial Sloan Kettering Cancer Center (MSKCC) or the International Metastatic RCC Database Consortium (IMDC) risk scores.

Taken together, these results demonstrated, as seen in the Kaplan–Meier plots, that the prognostic value of sCD206 was dependent on MSKCC and IMDC risk scores; however, with significantly increased HRs for intermediate‐risk patients by both MSKCC and IMDC.

### 3.6 sCD206 as a potential add‐on biomarker to improve the IMDC prognostic score

**FIGURE 4:**
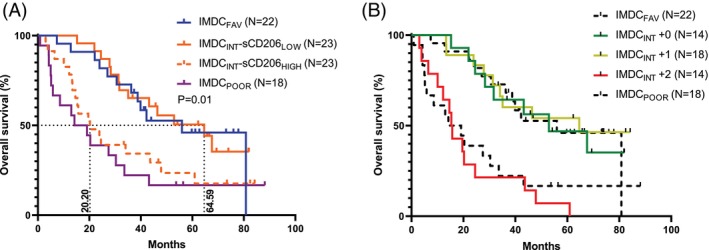
Survival analysis of mRCC patients stratified by IMDC risk groups and baseline levels of sCD206 and sCD163. (A) Overall survival of all patients stratified by the three IMDC risk groups. The IMDC_INT_ risk group was further divided using the median baseline level of sCD206 (0.283 mg/L) into two groups, defined as IMDC_INT_‐sCD206_LOW_ and IMDC_INT_‐sCD206_HIGH_. *p* value shows log rank test of IMDC_INT_‐sCD206_LOW_ versus IMDC_INT_‐sCD206_HIGH_ subgroups. Dotted lines including numbers written vertically on the *x*‐axis show median OS. (B) Overall survival of IMDC_INT_ patients subdivided into three groups based on the baseline median concentration of sCD206 and sCD163 in the IMDC_INT_ group. IMDC International Metastatic RCC Database Consortium; IMDC_INT_ + 0, both sCD206 and sCD163 below the median; IMDC_INT_ + 1, one of either sCD206 or sCD163 was above the median; IMDC_INT_ + 2, both above the median; sCD163, soluble CD163; sCD206, soluble CD206.

As mentioned above, the patients in the present clinical trial cohort were included based on their MSKCC risk score. However, the IMDC risk score is now the preferred system used for mRCC patients, and the above analyses indicated potential add‐on value, especially in the IMDC intermediate‐risk group. Hence, we analyzed whether sCD206 could improve the prognostic value of the IMDC score. The IMDC intermediate‐risk group was divided into two groups by the median baseline sCD206 value (0.283 mg/L): IMDC_INT_‐sCD206_LOW_ and IMDC_INT_‐sCD206_HIGH_. Interestingly, we observed a marked difference in median OS between these two groups (64.6 vs. 20.2 months, *p* = .01). The OS of the IMDC_INT_‐sCD206_LOW_ was comparable with the IMDC_FAV_ risk group, while the OS of the IMDC_INT_‐sCD206_HIGH_ was comparable to the IMDC_POOR_ group, demonstrated by no significant OS differences (both *p* > .40; Figure 4A).

In our previous study based on the same cohort of mRCC patients, we demonstrated that sCD163 had similar prognostic potential in IMDC_INT_ risk patients. Hence, to test whether the addition of both biomarkers would improve the prognostic potential further, we made a composite score based on both sCD206 and sCD163. If none of the two were above the median baseline value in the IMDC_INT_ group, a score of +0 were given, if only one was above the median value, a score of +1 were given, and if both were above the median, a score of +2 were given. The +0 and +1 groups demonstrated comparable OS curves, similar to IMDC_FAV_ patients (all *p* > .67), while the +2 subgroup had a markedly decreased OS, comparable to that of IMDC_POOR_ patients. There was a clear OS difference between the IMDC_INT_ +0 and +1 versus the +2 group (*p* < .001 for both), and with a median OS of 52.9 and 64.6 versus 15.5 months, respectively (Figure 4B).

Although not statistically significant (*p* = .12), the IMDC_INT_ +2 group demonstrated a tendency toward decreased OS compared to the IMDC_INT_‐sCD206_HIGH_ group.

Thus, a dichotomized model by sCD206 may add prognostic value for IMDC_INT_ risk patients, and a trend towards further improved performance was demonstrated by including both sCD163 and sCD206 in the same model.

## 4 DISCUSSION

To the best of our knowledge, this is the first report on sCD206 as a potential biomarker in mRCC patients, investigating associations with clinical–biochemical parameters, MSKCC and IMDC risk scores, and OS.

We demonstrate that high baseline levels of sCD206 were associated with poor outcomes, especially in the large group of patients with MSKCC and IMDC intermediate‐risk scores. No association was observed with the number of organs with metastases, but with the presence of liver metastases, which is in line with findings for sCD163 in the same cohort.^7^ Not surprisingly, strong associations between sCD206 and both CRP and sCD163 were found.

The baseline concentration of sCD206 was a prognostic factor in Kaplan–Meier analysis in the entire cohort when using both the 75th percentile and the upper reference limit (0.43 mg/L) cut‐off, which was also the case by Cox regression using sCD206 both as a continuous and a dichotomized variable. Indications of interaction were found between the HR of sCD206 and both MSKCC and IMDC risk scores. Stratification by MSKCC and IMDC risk scores demonstrated that the association between high baseline sCD206 and poor outcome was only observed in the intermediate‐risk groups. This indicates that a potential effect modification may be present between sCD206 and clinical risk scores and that the prognostic potential of sCD206 primarily seems to be present in the intermediate‐risk groups of both models. Importantly, the explanation of this observation may be found in the molecular signatures of RCC tumors. Studies exploring the genomic landscape of RCC have revealed two main subtypes; an angiogenic or an immunogenic signature.^17^, ^18^ Here, the fraction of patients with an angiogenic subtype decreased, and the immunogenic subtype increased when moving from the favorable to the intermediate‐risk group, and from the intermediate to the poor risk group. This was seen for both the MSKCC and IMDC models.^17^ Clinical data have supported these findings, demonstrating varying responses to TKIs (antiangiogenic therapy) and immunotherapy between the prognostic groups.^19^, ^20^ Hence, the potential effect modification of sCD206 by clinical risk groups may be attributed to the underlying distribution of molecular subsets across the clinical risk groups, which should be investigated further.

In the guideline from the European Society for Medical Oncology on RCC,^21^ the recommended treatment for patients with a favorable IMDC risk score is TKI in combination with anti‐PD‐1, while the combination of two checkpoint inhibitors (ipilimumab + nivolumab) is recommended for intermediate and poor‐risk patients, supporting the idea of different tumor drivers. Overall, both genetic and clinical data have suggested relevant differences in the tumor microenvironment (including immune cells) between risk groups. However, no risk group contains only angiogenic or immunogenic subsets,^17^ which may explain why combination therapy has been found most efficient by the latest studies. Therefore, it should be investigated further if broadly available and inexpensive blood‐based immune assays, for example, sCD163 and sCD206, may be used as biomarkers to indicate the molecular subtype, and hence guide the choice of treatment in the future. Specifically, if our results are reproduced in larger prospective (and preferably randomized) studies, such blood‐based assays may be used to re‐classify the large and heterogeneous intermediate‐risk group into the favorable and poor risk groups, respectively, and thus treat patients accordingly.

The role of CD206^pos^ TAMs has been studied in a range of cancer types, including gastrointestinal cancers, glioma, breast, and prostate cancer, with most studies showing an association between high infiltration of CD206^pos^ TAMs and poor survival.^22^ Few studies examining CD206 expression in tumors of mRCC patients exist, and generally include low numbers of patients and do not include patients in the favorable prognostic group.^23^, ^24^ In these papers, no statistically significant association was found between CD206^pos^ macrophage infiltration and outcome; however, the studies may be underpowered. The largest study examining CD206 expression in RCC is in non‐metastatic RCC (*n* = 185),^25^ where a high density of CD206^pos^ cells was associated with worse outcomes in both univariant and multivariant analysis. Overall, our knowledge in this field is limited by few studies examining CD206 (and CD163) expression in RCC, and thus, studies on larger cohorts are needed.

In other malignancies, CD163^pos^ TAMs have been widely investigated, and high tumor infiltration is mainly associated with poor patient out‐ comes.^26^, ^27^ In contrast, studies on CD206^pos^ TAMs are more limited, but show similar results as for CD163.^27^, ^28^ Studies on serum sCD206 in malignant diseases are even more limited, with reports from only a few cancers, including multiple myeloma^14^ and gastric cancer,^15^ where high sCD206 levels were associated with more severe disease and reduced OS in both studies, but only in the multiple myeloma study sCD206 was an independent marker of reduced OS.^14^, ^15^ Thus, future studies on sCD206 as a biomarker in malignant diseases are needed.

Limitations of the present study include an older treatment regimen for mRCC and the inclusion of patients in the clinical study based on the MSKCC instead of the more recent IMDC risk score. Furthermore, we have not investigated the underlying biological mechanisms by which high levels of sCD206 relate to worse clinical outcomes. We have previously demonstrated that human monocyte‐derived macrophages release sCD206 after stimulation by phorbol myristate acetate or adenosine triphosphate *in vitro,* and that this shedding was due to a non‐matrix metalloproteinase protease.^13^ CD206 is a well‐described marker of M2‐like macrophages, and the expression is thought to be upregulated on TAMs.^29^ Our hypothesis is that serum levels of sCD206 may reflect the dynamics of the tumor immune microenvironment. Studies are ongoing with a collection of samples to investigate associations between tumor and serum levels of macrophage‐activation biomarkers (NORDIC‐SUN trial, NCT03977571). However, such samples were not available for inclusion in the present study.

In conclusion, we have demonstrated that sCD206 holds potential as a new biomarker for patients with mRCC, with higher levels being associated to more severe disease, and with possible prognostic impact especially for patients with intermediate‐risk disease.

If the present results are reproduced in newer, larger, and independent patient cohorts, sCD206 may be integrated with the IMDC risk score to improve prognostic precision for patients in the large intermediate‐risk group. Hereby, clinicians may be enabled to make more qualified choices about treatment for newly diagnosed mRCC patients. Thus, our findings could be a step toward personalized treatment decisions. To reach this goal, further studies are needed including randomized trials with biomarker‐guided treatment.

## AUTHOR CONTRIBUTIONS

**Kasper Munch Lauridsen:** Conceptualization; data curation; formal analysis; funding acquisition; investigation; methodology; project administration; visualization; writing – original draft. **Holger Jon Møller:** Methodology; resources. **Mie Wolff Kristensen:** Data curation; project administration. **Niels Fristrup:** Investigation; resources. **Frede Donskov:** Conceptualization; data curation; funding acquisition; investigation; methodology; project administration; resources; supervision. **Marianne Hokland:** Conceptualization; funding acquisition; methodology; project administration; resources; supervision. **Morten Nørgaard Andersen:** Conceptualization; data curation; formal analysis; funding acquisition; investigation; methodology; project administration; resources; supervision; visualization; writing – original draft.

## CONFLICT OF INTEREST STATEMENT

Niels Fristrup has received speaker honoraria from AstraZeneca, MSD, and Ipsen. Other authors declare no conflict of interest.

## ETHICS STATEMENT

The Central Denmark Region Committee on Health Research Ethics approved the DaRenCa‐1 clinical trial (M‐20070190), and the present study (1‐10‐72‐162‐23). All patients provided signed consent forms before inclusion in the clinical trial.

## Supporting information


**Data S1.** Supporting Information.

## Data Availability

The data that support the findings of this study are available from the corresponding author upon reasonable request.
