# Supplementary material for: Soluble CD206 in metastatic renal cell carcinoma: Relation to clinical–biochemical parameters and patient outcome
Source: Int J Cancer. 2024 Sep 25;156(4):875–85. doi: 10.1002/ijc.35194 (PMC11661521; doi:10.1002/ijc.35194)
Supplement: Supplementary file 1 — Data S1. Supporting Information. [file IJC-156-875-s001.pdf]

Supplementary materials for

Full title: Soluble CD206 in Metastatic Renal Cell Carcinoma: Relation to Clinical-Biochemical Parameters and Patient Outcome.

Author list: Kasper Munch Lauridsen, Holger Jon Møller, Mie Wolff Kristensen, Niels Fristrup, Frede Donskov, Marianne Hokland, Morten Nørgaard Andersen

Table of contents

Supplementary Figure 1:	Overall survival by serum sCD206 after stratification based on MSKCC risk groups.
Supplementary Figure 2:	Overall survival by serum sCD206 after stratification based on IMDC risk groups.

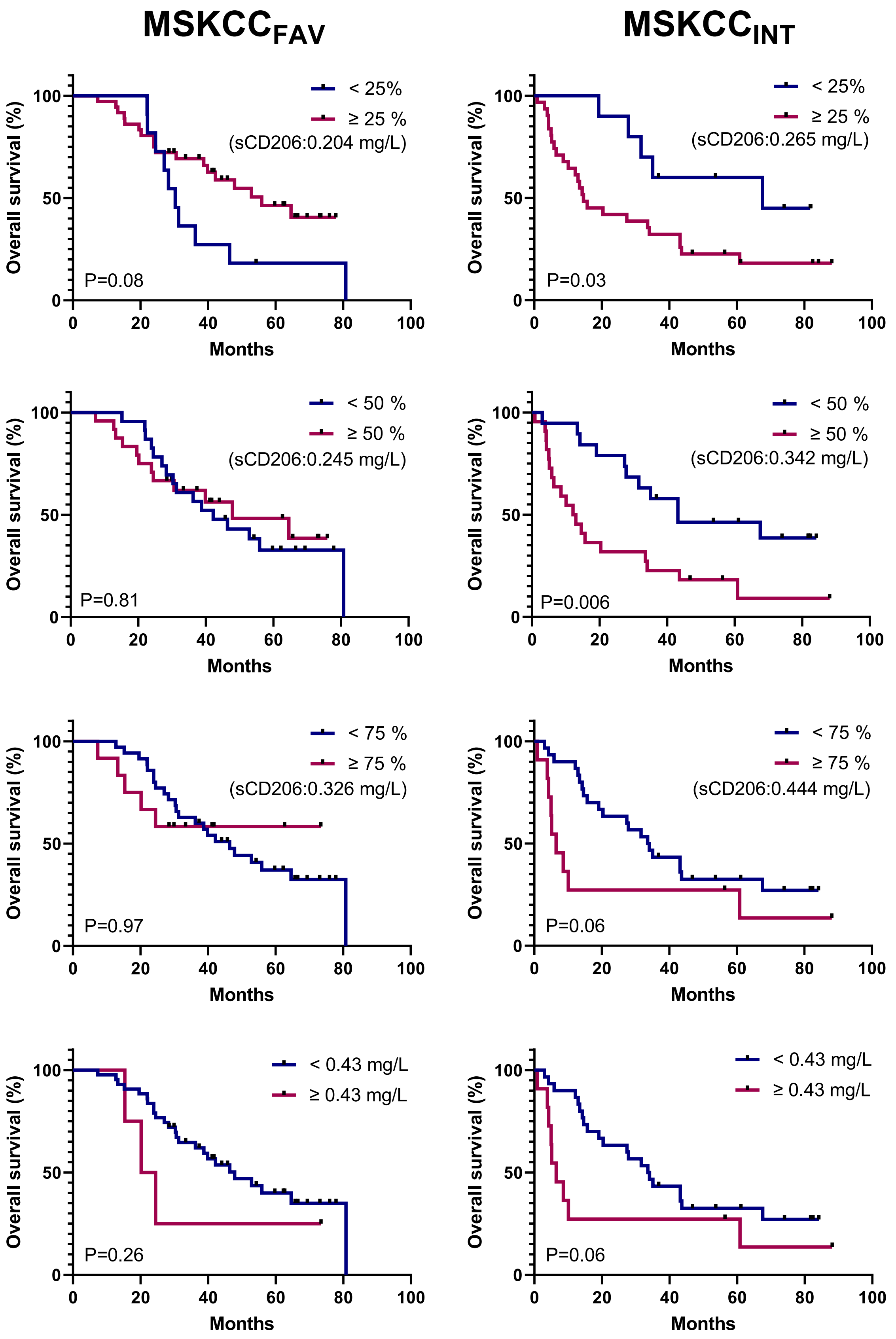

Supplementary Figure 1:
Overall survival by serum sCD206 after stratification based on MSKCC risk groups.

Left and right columns show patients with $MSKCC_{FAV}$ and $MSKCC_{INT}$ risk, respectively.
Overall survival is depicted as months on the X-axes. The separator value of serum sCD206 (in mg/L) used for the binomial categorization is shown in parentheses on each graph. P-values by log-rank test. Censored patients are annotated with a rectangle on top of the line. 0.43 mg/L is representative of the upper normal range of sCD206.
Only four of $MSKCC_{FAV}$ patients were above the upper reference limit (N=4), so this analysis is related with has a high degree of uncertainty.
For the $MSKCC_{INT}$, the 75th percentile and the upper reference limit resulted in the same dichotomization of the patients.

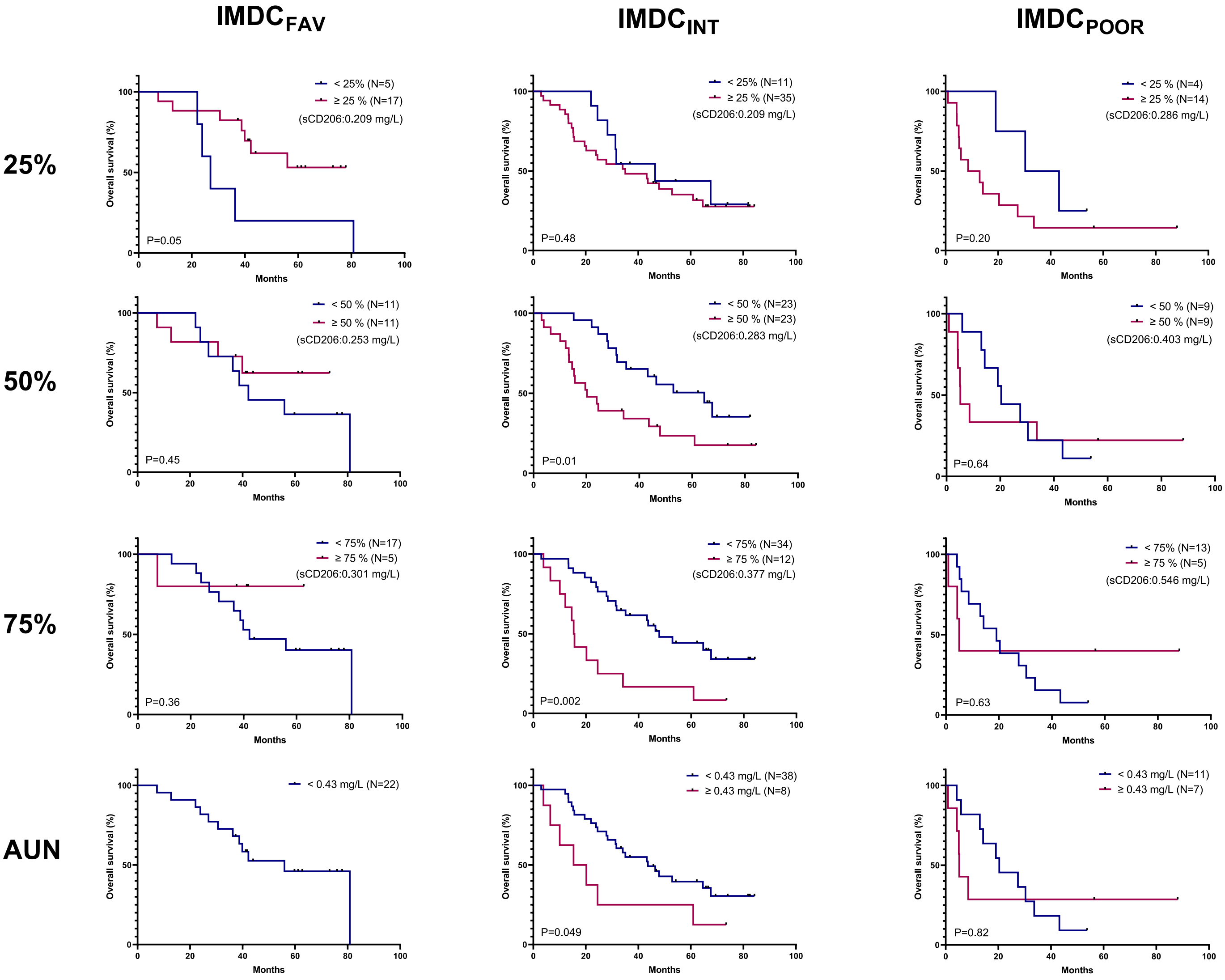

Supplementary Figure 2:
Overall survival by serum sCD206 after stratification based on IMDC risk groups.
Left, middle, and right columns show patients with IMDC$_{FAV}$, IMDC$_{INT}$, and IMDC$_{POOR}$ risk, respectively. Overall survival is depicted as months on the X-axes. Each risk group was stratified by the 25th, 50th, 75th percentile, as well as the upper normal range (AUN, 0.43 mg/L). The separator value of serum sCD206 (in mg/L) used for the binomial categorization is shown in parenthesis on each graph. P-values by log-rank test. Censored patients are annotated with a rectangle on top of the line. None of IMDC$_{FAV}$ patients were above the upper reference limit.
IMDC: International Metastatic RCC Database Consortium. sCD206: Soluble CD206.
